# Pediatric Cardiovascular Multiscale Modeling using a Functional Mock-up Interface

**DOI:** 10.1007/s13239-024-00767-6

**Published:** 2025-01-06

**Authors:** Ellen E. Garven, Ethan Kung, Randy M. Stevens, Amy L. Throckmorton

**Affiliations:** 1 School of Biomedical Engineering, Science and Health Systems, Drexel University, 3141 Chestnut Street, Rm. 718, Philadelphia, PA 19104 USA; 2 Department of Mechanical Engineering, Department of Bioengineering, Clemson University, Clemson, SC USA; 3 St. Christopher’s Hospital for Children, Philadelphia, PA USA; 4 Pediatrics, College of Medicine, Drexel University, Philadelphia, PA USA

**Keywords:** Computational modeling, Functional mock-up interface, Multiscale modeling, Single ventricle, Univentricular

## Abstract

**Purpose:**

Computational models of the cardiovascular system continue to increase in complexity. As more elements of the physiology are captured in multiscale models, there is a need to efficiently integrate subsystems. The objective of this study is to demonstrate the effectiveness of a coupling methodology, called functional mock-up interface (FMI), as applied to multiscale cardiovascular modeling.

**Methods:**

The multiscale model is composed of two subsystems: a computational fluid dynamics (CFD) model coupled to a lumped parameter model (LPM). The LPM is packaged using the FMI standard and imported into the CFD subsystem using an FMI co-simulation architecture. The functionality of an FMI coupling was demonstrated in a univentricular parallel circulation by means of compatible tools, including ANSYS CFX and Python. Predicted pressures and flows were evaluated in comparison with clinical data and a previously developed computational model.

**Results:**

The two models exchanged pressure and flow data between their boundaries at each timestep, demonstrating sufficient inter-subsystem communication. The models recreated pressures and flows from clinical measurements and a patient-specific model previously published.

**Conclusion:**

FMI integrated with ANSYS CFX is an effective approach for interfacing cardiovascular multiscale models as demonstrated by the presented univentricular circulatory model. FMI offers a modular approach towards tool integration and is an advantageous strategy for modeling complex systems.

## Motivation

There is considerable ongoing investment in the innovation of new technology-driven and discovery-driven therapies to transform outcomes for pediatric patients with acquired or congenital cardiovascular diseases. Realization of many milestone achievements in pediatric cardiovascular innovation is motivated by significant advancements in computational modeling for treatment planning and care management. Computer technology, parallel processing capabilities, and advancements in numerical methods have facilitated the ability to conduct sophisticated, complex modeling of pediatric cardiovascular disease. The adoption of these techniques are further cemented by the issuance of new guidelines from the US FDA for modeling and simulation in medical device regulatory submissions [1].

In many cardiovascular models, hemodynamics are simulated using computational fluid dynamics (CFD) [2]. Patient-specific imaging is leveraged to develop 3D geometries of the anatomy, and the blood flow within the region is simulated and analyzed; whether to understand disease progression or the impact of a treatment intervention, the study of an individual’s hemodynamics is fundamental to therapeutic progress. While CFD provides a detailed analysis of the fluid mechanics, these simulations are computationally complex and therefore the geometry is often limited to a local region of the anatomy. However, additional techniques are required in order to capture the influence of the rest of the cardiovascular system on the physics within the local region. Multiscale modeling approaches have addressed this issue by coupling CFD analyses of the local geometry to lumped parameter models (LPM) that capture the upstream and downstream effects using a fluidic-electrical analogy [3]. The multiscale approach has been well documented in blood flow problems and has been meaningfully implemented in many pediatric cardiovascular studies [4, 5]. Pediatric multiscale models have evaluated novel surgical configurations [6], simulated virtual surgery [7], and studied diseased physiology under conditions like exercise [8].

Groups have previously developed cardiovascular multiscale models using a range of tools, including the use of commercial CFD software as well as custom codes [4, 9]. While these platforms have a rich history demonstrating their effectiveness, there remain barriers to the implementation of more complex multiscale and multiphysical models that incorporate additional physiological processes. For instance, whole heart simulations that couple the electrophysiology at the cellular level, to the solid mechanics of the ventricular walls, and to the fluid dynamics across the circulatory system require significant computational efforts [10]. As the number of individual components in a mathematical model increases, there is a greater need for efficient coupling strategies between the component subsystems.

Drawing from computational approaches in other fields, a Functional Mock-up Interface (FMI) is a unique approach to address this coupling challenge. FMI is a set of standards developed as a coupling framework used for complex modeling systems. It defines a standardized interface for the dynamic exchange of data between subsystems that are simulated by different software tools. Originally designed for model-based development in the automotive industry, FMI is a relatively new methodology that is gaining traction in multiple fields of engineering, with recent applications in airflow, thermal, and nuclear analyses [11–13]. From a systems engineering standpoint, FMI is advantageous because it allows for the modular integration of multiple model components; this is valuable because individual subsystems can be developed, tested, and verified in isolation before their integration in a more complex environment. Tool independence and modularity enables greater efficiency in future simulation efforts, where individual subsystem models can be readily repurposed in new analyses. Instead of necessitating an all-in-one software or completely custom code, individual subsystems can be simulated using different software tools, each chosen to best capture the physics of the subsystem or at the preference of the individual research group. Many popular simulation software systems have already implemented features compatible with FMI, including tools from Siemens, ANSYS, MathWorks, and Dassault.

Due to its advantages in modeling complex systems, FMI is well-suited for cardiovascular applications given the number of multiscale and multiphysics models that can describe the system. In our review of the literature, we found only one previous analysis that used an FMI approach, where a finite element mechanical model of the ventricular walls (LS-DYNA) was coupled to a lumped circulatory model (Simulink) [14]. While this model was developed for mechanical analysis in the context of ischemic heart disease, the same coupling methodology can be applied to other cardiovascular applications.

In particular, pediatric populations are ideally suited for the versatile nature of an FMI modeling approach. Infants and children have dynamic biopsychosocial, growth, and developmental complexities that progress from birth to early adulthood. The modularity of FMI allows for the seamless integration of various subsystems, establishing a dynamic platform capable of accommodating an evolving physiology. The interchangeability of subsystems facilitates in the capture of variations in illnesses, diseased states, growth rates, and treatment strategies. As an alternative to other coupling methodologies, an FMI coupling is a modularized and adaptable system, well-positioned to address the distinct challenges and complexities inherent in this vulnerable patient population.

In this study, we integrate a multiscale framework for a pediatric cardiovascular model via an FMI co-simulation coupling. We demonstrate the methodology of the coupling using ANSYS CFX and evaluate its performance in a study of the hemodynamics in a patient with a univentricular parallel circulation. The successful integration of model subsystems is shown through the exchange of physiological data between the boundaries.

## FMI Co-Simulation of a Univentricular Parallel Circulation

### Clinical Significance & Motivation

**Fig. 1:**
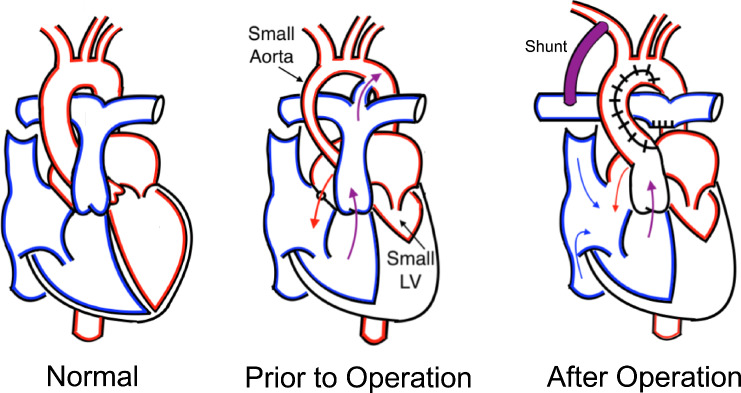
Illustration of a normal heart (left) in comparison to a heart with common single ventricle features (middle), including an abnormally small left ventricle (LV) and aorta. After the Norwood (right) a shunt connects the innominate and right pulmonary arteries in a parallel circulation

Single ventricle malformations are a severe form of congenital heart defect in which one of the two normal ventricles of the heart is missing or malformed. Infants born with single ventricle defects require cardiac surgery shortly after birth; in the first of several staged palliative surgeries, the Norwood procedure establishes a univentricular circulation that delivers blood to both the lungs and the body (Fig. 1). This is achieved through the implantation of an artificial graft called a shunt, which redirects a portion of the blood flow from the aortic arch and into the pulmonary artery; the blood is pumped in a parallel circuit to both the systemic and pulmonary circulations. The Norwood remains the riskiest stage of the single ventricle palliation plan, and the associated risk extends throughout the four to six month period that the shunt remains in place before the next surgical stage [15, 16]. Despite advances in the field and in overall patient care, management of the univentricular circulation remains challenging and is associated with elevated rates of morbidity and mortality.

A parallel circulation results in a mixture of oxygenated and deoxygenated blood in the ventricle. This arrangement can only sustain the patient if the mixture is properly balanced, with adequate blood saturation and sufficient body perfusion. With the shunt being the primary source of pulmonary flow, the conditions surrounding the shunt have a significant impact on the hemodynamic balance.

Computational modeling is a valuable approach that can help evaluate the hemodynamics in this vulnerable patient population and thereby reduce the risks of adverse outcomes. Previous computational models of this surgical stage have been utilized to reproduce patient-specific hemodynamics, compare different shunt configurations, and conduct pre-surgical planning and optimization [17–19]. These techniques have spanned computational models composed of CFD-only, LPM-only, and multiscale approaches. Because of the number of computational studies surrounding this univentricular circulation and the continued clinical need to improve outcomes, we chose to demonstrate the FMI coupling in this clinical scenario.

### FMI Coupling

FMI is a standard designed to simplify the integration of models operating with different simulation software systems. It establishes a set of specifications that define a leader/follower architecture between coupled subsystem models. The follower subsystem is packaged into a file called the functional mock-up unit (FMU), which is linked with the leader subsystem through the standardized interface.

An FMU is a zipped file containing the follower model abstracted into a set of XML files, binaries, and C code. While a direct implementation of the specification can be used to create the FMU, many software packages have already incorporated these features, and several independent tools have also been published. The XML files within the FMU define the elements of the model in a standardized format, including variables for time, inputs, outputs, and parameters. The equation describing the computations are implemented using C to provide the most flexibility and compatibility with a wide range of systems. The FMU is also packaged with its own numerical solver in a co-simulation interface.

The leader subsystem controls the progression of the simulation; it sets the inputs, gives the command to step forward in time, and reads the subsystem outputs. Elements exchanged at the interface can be scalar or multi-dimensional arrays and exist in a number of data types, with or without corresponding physical units. The FMI standard defines the encoding of the model and its parameters, ensuring interoperability across different simulation environments while allowing each simulation environment to manage the interfacing data stutructures to its own ideal.

**Fig. 2:**
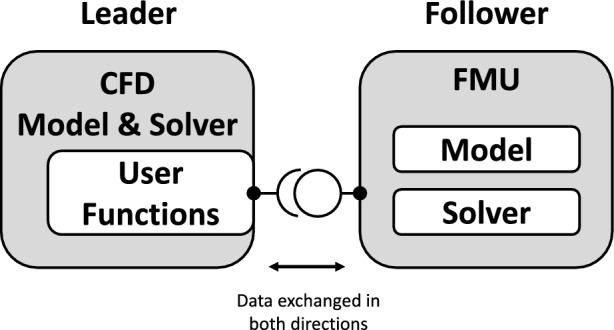
In a co-simulation structure, the follower subsystem is packaged into a functional mock-up unit (FMU) and imported into the leader subsystem. The leader accesses the FMU through a standardized interface

As applied to a pediatric cardiovascular multiscale model, the CFD model was designated as the leader, and the LPM as the follower, as shown in Fig. 2. From the list of software compatible with importing co-simulation FMU, we selected ANSYS CFX, which is in line with our ongoing research in pediatric medical devices, pediatric mechanical circulatory support, and blood shunts [20, 21]. The LPM was developed as a system of ordinary differential equations and packaged into an FMU. Before it was imported into the CFD software, the FMU was validated against the FMI standard using the FMI Compliance Checker, developed by Modelon AB [22].

**Fig. 3:**
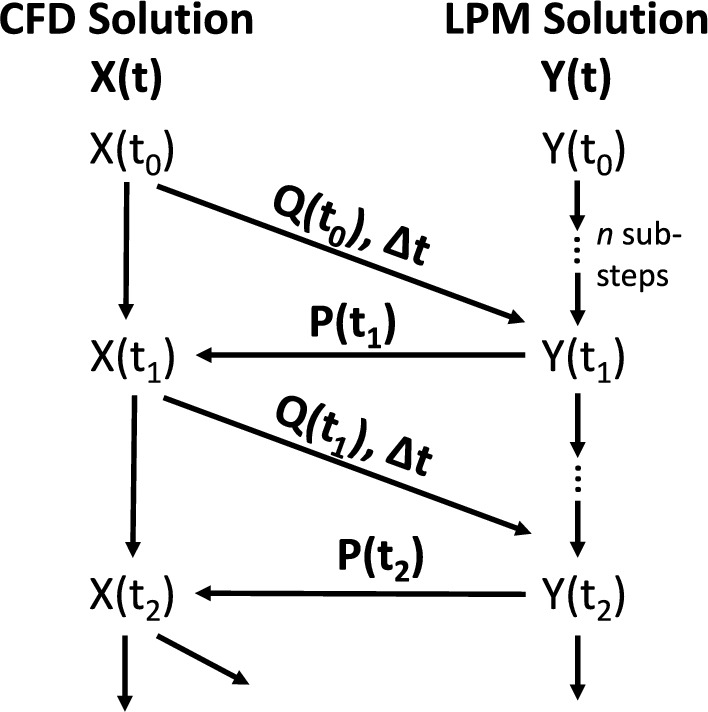
Scheme for advancing the numerical solutions of the CFD (X) and LPM (Y) subsystems. Flows (Q) and pressures (P) at each boundary are exchanged at each timestep ($\Delta t$)

The multiscale model interfaced the two subsystem models with an exchange of complementary boundary condition data; the boundary conditions of one subsystem were provided by the solution of the other in a tightly-coupled scheme [3, 10, 23, 24]. As shown in Fig. 3, the forward step in time was initiated and controlled by the CFD software, which ran with an adaptive time-stepping method. Once the CFD subsystem was iterated to a sufficient stability for that timestep, the resulting flow rates at the boundaries were passed to the LPM. For LPM solution stability, the CFD timestep was subdivided within the LPM and a fourth order Runge-Kutta method was iterated. The LPM timestep was subdivided to be less than or equal to 100 μs, a threshold chosen based on prior reports of LPM solution stability [7].

### Subsystem Model Development

**Fig. 4:**
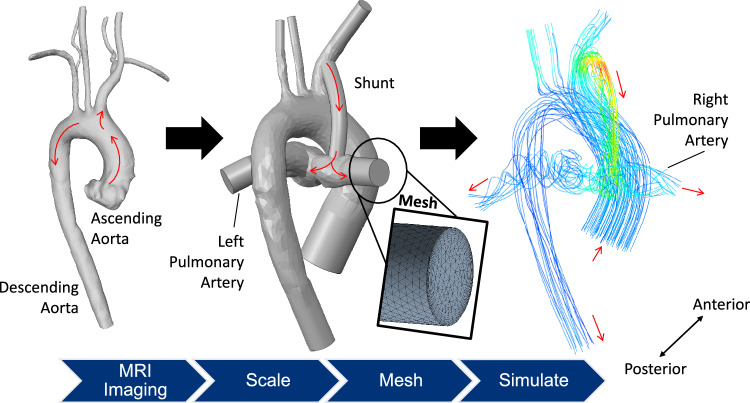
Workflow to generate the CFD subsystem from anatomical imaging, geometry development, meshing, and simulation. Red arrows indicate direction of ideal flow

For the CFD subsystem, a model of the parallel univentricular anatomy that we previously developed was utilized, for details see Garven et al. 2022 [21]. Briefly, anatomical data from a healthy adult was processed into a 3D geometric model using image processing and computer-aided design software (Fig. 4). The geometry was scaled to neonatal dimensions and isolated to the region of interest, including the aortic arch, arterial branches, and left and right pulmonary arteries. A modified Blalock-Taussig shunt (MBTS) was added between the innominate and right pulmonary arteries based on surgical sketches, the placement of which was reviewed by a pediatric cardiac surgeon. The shunt diameter was 3.5 mm, reflecting the diameter most commonly implanted.

The anatomical geometry was imported into ANSYS Mesh, where a mesh composed of millions of tetrahedral elements was generated. For each mesh created, element quality was assessed using standard mesh quality metrics including skewness (average < 0.33, maximum < 0.95) and aspect ratio (average < 50, maximum < 200). To sufficiently resolve the boundary layer, 10 inflation layers corresponding to a y +  ≤ 1 were added to each domain. The density of the mesh was evaluated in a grid-independence study, whereby the solution stability of iteratively finer grids was tested. Resulting pressures and velocities for each mesh were evaluated in comparison to those predicted by the finest, highest density mesh. Meshes with < 3% difference across all metrics were identified as grid-independent. The grid-independent mesh with the fewest number of elements was selected for further analysis, and the final mesh implemented contained 3.4 million elements.

ANSYS CFX (2019 R3) was used to simulate the system using a Reynolds-Averaged Navier-Stokes solver. Blood was assumed to be Newtonian with a density of 1050 kg/m^3^ and a viscosity of 3.5 cP. While blood is known to be non-Newtonian at low shear rates, the Newtonian assumption is commonly made in cardiovascular literature, particularly in studies of the aorta and other large vessels, where shear rates below this threshold are unlikely to occur [3, 25]. Due to the pulsatility in the aorta, the critical Reynolds number for turbulent flow was estimated using the relationship described by Nerem et al. [26]. Using the diameter of the ascending aorta as the characteristic length, the critical Reynolds number was calculated as 2305. Turbulent flow was assumed given that the peak Reynolds number was 2465 based on our expected velocities. Blood flow in the aorta is often assumed to be transitional, and studies have demonstrated the influence of turbulence near the walls on variables such as the wall shear stress [27, 28]. Thus, the k-ω SST turbulence model [29] is well-suited to capture both the near-wall and transitional flow regimes of the aorta [30–32]. The CFD solver was executed using an adaptive timestep and a second order backward Euler scheme.

For the LPM subsystem, a model of the circulatory system was adapted from a previously established and validated LPM [7, 33]. This model was modified to complement the 3D anatomical geometry, and was described by a system of ordinary equations that captured the closed-loop cardiovascular physiology of the patient outside of the CFD model. The model equations were written in Python, and a fourth order Runge-Kutta method was employed as the numerical solver.

Since the LPM was designed as the follower subsystem in the FMI coupling, the timestep was set and controlled by the CFD solver. However, the differences in solvers between the CFD and LPM subsystems necessitated different scales in the timestep to obtain mathematical stability. Because of this difference, the adaptive timestep set by ANSYS CFX was imported and subdivided (Fig. 2) such that the LPM-internal timestep was less than or equal to 100 μs. This threshold was utilized in the previously developed LPM and verified in the new multiscale framework for solution stability []. The LPM subsystem was packaged as an FMU (version 2.0) for co-simulation export using the PythonFMU framework [34]. The packaging and structure of the FMU was verified to be compliant with the FMI standard using the Modelon FMI Compliance Checker [22].

### Executing an FMI Multiscale Simulation

**Fig. 5:**
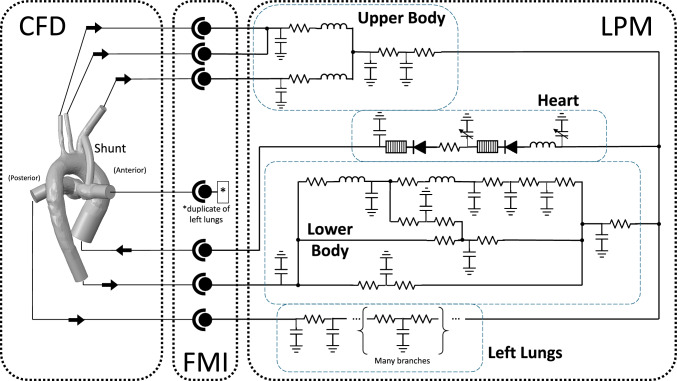
Schematic of the FMI multiscale coupling between the CFD and LPM subsystems. Data is exchanged at each boundary

A schematic of the complete multiscale model and interfacing conditions is shown in Fig. 5. The LPM was imported into ANSYS CFX as an FMU. Values exported from the FMU were linked to pressure boundary conditions in the CFX model. Expressions for the mass flow at each boundary were imported into the FMU, along with the current value of the adaptive timestep. The simulation was executed over eight cardiac cycles and data from the final cycle was used for analysis.

Our goal was to demonstrate the effectiveness of FMI in exchanging physiologically relevant data between a CFD model and a LPM of the circulation. Manual tuning of LPM parameters was conducted to better reproduce patient-specific clinical measurements. These values were assessed by averaging over a cardiac cycle and calculating the percent differences against clinical measurements, which were previously recorded by Schmidt *et al*. 2017 for a Norwood patient [33]. In order to assess the time-dependent behavior, the results of the multiscale model were compared to waveforms recorded by the LPM from which our system was adapted [7, 33]. Pressures and flows were qualitatively compared over a cardiac cycle at several anatomical landmarks. The root-mean-squared error (RMSE) was calculated to further compare the time- dependent behavior between the two datasets; this error was normalized against the range of that variable in the LPM waveform and expressed as a percentage.

## Results

The simulation ran successfully over many cardiac cycles. Averages across the final cardiac cycle were calculated for several variables and reported in Table 1. These averages were compared to a set of patient-specific clinical measurements previously described and the percent difference calculated [33]. The multiscale model had a slightly larger volume of flow throughout the system but maintained a reasonable distribution in the ratio of pulmonary to systemic flow (Qp/Qs). The differences between the averaged simulation results and the clinical measurements were below 10% for each variable recorded.

**Table 1:** A comparison of the model simulated values against clinical data for parameters including the cardiac output (CO), upper body flow (QUB), descending aorta flow (QDAO), shunt flow (QSH), aortic pressure (PAO) and pulmonary pressure (PPUL)

Parameter	Units	Clinical Value	Model Value	Difference (%)
CO	mL/s	21	22.01	4.8
Q_UB_	mL/s	5.6	5.79	3.4
Q_DAO_	mL/s	5.7	6.04	5.9
Q_SH_	mL/s	9.7	10.18	4.9
Qp/Qs	-	0.9	0.86	4.4
P_AO_	mmHg	52	54.6	5.0
P_PUL_	mmHg	12	11.9	0.8

**Fig. 6:**
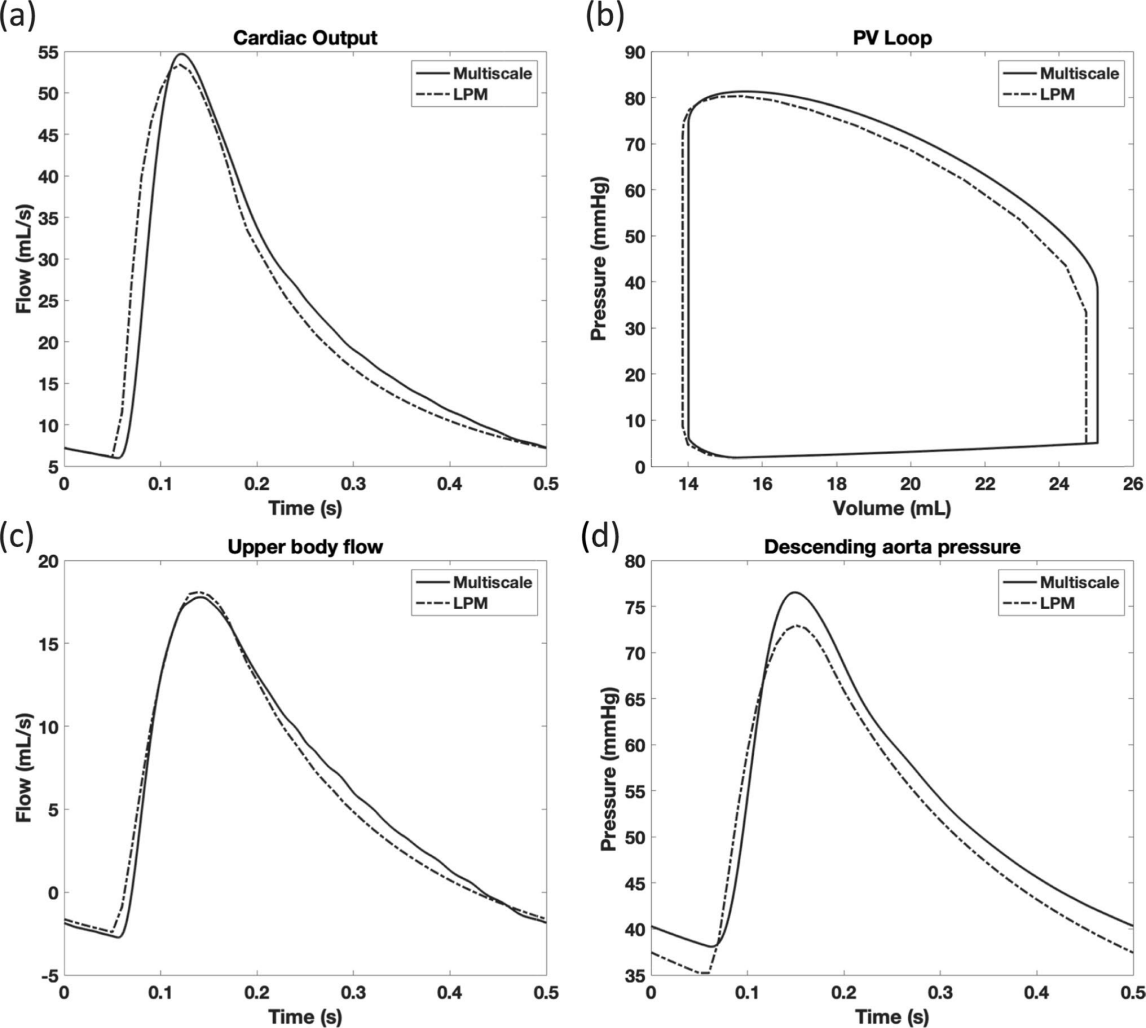
The multiscale model compared against the LPM-only model for the cardiac output (**a**), the ventricular pressure-volume loop (**b**), the upper body flow (**c**), and the descending aorta pressure (**d**)

Because the model was adapted from a previously established and validated LPM [7, 33], the results were compared to that LPM-only model to establish the physiological relevance given the changes that were made in the multiscale implementation. Key physiological time-dependent waveforms were compared to the LPM-only model, samples of which are shown in Fig. 6a-b. The cardiac output in the multiscale system followed the LPM-only data, with a slightly higher peak value and some shifting in time. This difference was quantified by the normalized RMSE, which was calculated to be 8.5%. The ventricular pressure-volume loop showed an offset during systole, which is indicative of a greater amount of preload in the multiscale model than in the LPM-only model (normalized RMSE for pressure was 0.8%, and volume was 2.5%).

With the goal of testing the coupling methodology, data exchanged between the two models were monitored for numerical stability and successful input/output with the other model. In Fig. 6c-d, two examples of the pressures and flows at boundaries between the CFD and LPM models on the systemic side were shown; pressures were outputted from the LPM and imported into CFD, whereas mass flow rates were outputted from the CFD model and imported into the LPM. Consistent with the other pressures in the multiscale data, these pressures were higher than expected and the normalized RMSE was 7.5% The flow in the upper body arteries followed the reference waveform more closely, with an error of 4.1%. Similar behavior was found in the other variables of the multiscale model.

## Discussion

In this paper, we demonstrated the implementation of an FMI co-simulation coupling as an approach for pediatric cardiovascular modeling. As shown within the parallel univentricular physiology, the coupling methodology interfaced two subsystems that simulated physiologically relevant pressure and flow waveforms in a closed-loop circulatory model; a 3D model of the dynamics within an anatomical geometry was simulated in ANSYS CFX and interfaced to a lumped model of the vasculature in Python. The system reproduced the given set of clinical data and the qualitative time-dependent behavior of previous models [33]. The normalized RMSE was high, however additional tuning of the system would minimize the remaining differences; these errors are unlikely to impact the functionality of the FMI methodology to successfully exchange physiological information between models.

The FMI modeling methodology offer advantages due to its ease of integration and interoperability. As simulation needs and techniques continue to rapidly evolve, the adaptability of the simulation tools being utilized is increasingly crucial for efficient development.The FMI approach allows researchers to select the most appropriate tool for specific analyses, without the need to integrate new features into existing software. This integration ability is especially appealing when considering future work, such as implementing assist devices, studying fluid-structure interactions, or examining other multi-physical models of the electrophysiology. By leveraging the strengths of established software systems, FMI can help make the design and development of therapeutic devices more effective.

Alternatively, previous multiscale studies using CFX relied on user functions, which required a significant depth of knowledge in the software [6]. On the other hand, FMI is well-documented in its setup and function, and independent tools can be used to aid in the verification process. This makes FMI a more accessible avenue for implementing multiple model systems.

A limitation of this approach is that it requires the underlying code to support FMI co-simulation; however, several popular commercial CFD codes, such as ANSYS CFX, STAR-CCM + , and OpenFOAM, have already incorporated these features. It should be noted that these commercial codes are typically only compatible with importing FMUs (and cannot themselves be exported as FMUs), which limits the potential for broader software coupling and integration. To aid in assessing compatibility, the FMI standard maintains^1^ a list of over 200 compatible tools, which can be sorted by available functionality. While there are other limitations to the multiscale model geometry and implementation, the underlying assumptions were appropriate for the specific goal of evaluating the FMI coupling. In comparison to other hemodynamic-specific modeling frameworks, features such as backflow stabilization remain as limitations in our current approach and will be addressed in future work [35].

While FMI has been increasingly adopted across industries, its implementation is still relatively new, and its features are still under development, especially in commercial codes. We experienced a compatability issue related to software licenses in our initial implementation, despite the chosen software system appearing on the list of supported tools. It is possible that the issue has been resolved in the time since, however we ultimately selected a different tool for our purposes. The process of isolating these issues was aided by independent verification tools such as the FMI Compliance Checker [22].

There remain significant challenges to the treatment of pediatric cardiovascular diseases, and computational models will continue to serve a role in evaluating existing and novel treatment strategies. By creating efficient modeling platforms, the FMI approach could advance further computational research avenues by streamlining the integration of many model subsystems. With a flexible architecture, previously developed patient-specific models could be leveraged and adapted in studies of other patients, conditions, and therapeutic strategies.

## Conclusion

In conclusion, a cardiovascular multiscale model was developed and assessed in a study of the parallel univentricular physiology. The model sufficiently reproduced pressures and flows, demonstrating the effectiveness of the FMI implementation in representing the physiology and facilitating communication between the two model subsystems. To our knowledge, this is the first study to establish an FMI coupling for a pediatric cardiovascular model. The modularity and integration ability inherent to FMI are advantageous features and this methodology could be valuable for future cardiovascular translational applications.

## Data Availability

The data that support this study are available upon reasonable request.

## Footnotes

1 https://fmi-standard.org/tools/
